# GPs’ experienced challenges and strategies for supporting patient self-management in disease management programs for type 2 diabetes mellitus and coronary heart disease - a qualitative study

**DOI:** 10.1186/s12875-025-02896-w

**Published:** 2025-07-12

**Authors:** Larisa Pilic, Kira Molkentin, Alina Herrmann, Marcus Redaèlli, Lisa Kupsch, Lion Lehmann, August-Wilhelm Bödecker, Beate Sigrid Müller, Stephanie Stock, Stefan Wilm

**Affiliations:** 1 Institute of General Practice, Faculty of Medicine and University Hospital Cologne, University of Cologne, Kerpener Str. 62, 50937 Cologne, Germany; 2 Institute of Global Health, Heidelberg University, Heidelberg, Germany; 3 Institute of Health Economics and Clinical Epidemiology (IGKE), Faculty of Medicine and University Hospital Cologne, University of Cologne, Cologne, Germany; 4 Institute of General Practice, Heinrich Heine University Düsseldorf, Düsseldorf, Germany

**Keywords:** Patient motivation, Self-management, Disease management program (DMP), Type 2 diabetes mellitus (T2DM), Coronary heart disease (CHD), General practice

## Abstract

**Background:**

Effective self-management (SM) is essential for improving health and preventing severe complications in patients with lifestyle-related chronic conditions, such as type 2 diabetes mellitus (T2DM) and coronary heart disease (CHD). Thus, enhancing patients’ SM through self-management support has become an integral part of chronic care programs worldwide. However, information on the current focus on SM in German disease management programs (DMPs) is very limited. The aim of this study was to understand general practitioners’ (GPs’) experiences and strategies for promoting SM.

**Methods:**

An exploratory qualitative design was selected, resulting in the conduct of five focus group discussions with 20 GPs in August and September 2020. The GPs were recruited from the teaching practices of the University Hospital Cologne through purposive sampling. Their experiences and opinions on SM were assessed in questions blocks using a semi-structured interview guide and analysed by a multi-professional team employing Kuckartz’ method of qualitative content analysis.

**Results:**

The focus group discussions were structured around three main categories: (1) GPs’ perceived patient SM in the DMPs for T2DM and CHD, (2) GPs’ perceptions of factors influencing patient motivation for SM, and (3) strategies fostering patient motivation for SM. Discussions revolved around patients’ motivation and capabilities to implement a sustainable SM in their daily lives. Many GPs followed a patient-centered approach to foster SM in their patients, considering various challenging individual, social and institutional factors that influence SM in chronically ill patients.

**Conclusion:**

GPs regularly support their patients’ SM as a routine part of the DMP through ongoing consultations and education. However, they face several challenges in facilitating a sustainable patient SM, which requires support by additional and standardized measures beyond the current DMP care to be effective.

**Supplementary Information:**

The online version contains supplementary material available at 10.1186/s12875-025-02896-w.

## Background

In Germany, the eight most common causes of death are noncommunicable diseases, with coronary heart disease (CHD) being the leading cause of death in Germany and globally [1]. Furthermore, life expectancy in Germany is below average compared to other OECD countries, which has been attributed to an insufficient focus on prevention at the primary care level, especially for cardiovascular diseases [2]. This underlines the importance of high-quality primary care for people with noncommunicable diseases.

In 1998, Wagner et al. developed the Chronic Care Model (CCM) as a framework for improving the quality of primary care for patients with chronic diseases [3]. In Germany, structured care programs for specific chronic diseases are operationalized in disease management programs (DMPs). The four current DMPs in primary care for standardized treatment of type 2 diabetes mellitus (T2DM), CHD, asthma and COPD were implemented in general practice between 2002 and 2005 [4]. The two largest DMPs, covering approximately 4.5 million patients with T2DM and around 1.9 million patients with CHD in 2023 [5], appear to be effective in terms of treatment outcomes and align with guidelines and other chronic care parameters [6–8]. However, the evidence on the effectiveness of German DMPs is characterized by great heterogeneity [9–15]. On the one hand, significantly more studies have been published on the effectiveness of the DMP for T2DM than on the effectiveness of other DMPs. On the other hand, the evidence on the effectiveness of the DMP for T2DM is inconclusive due to the low methodological quality of some studies. This includes inconsistent data collection methods, unsuitable study designs, and selection bias, which limits the ability to summarize statements on the effectiveness of the DMP [9–10]. Some evaluation studies employing a robust design have shown that participation in the DMP for T2DM measurably improved patients’ health, with positive effects on mortality as well as on regular blood pressure and blood glucose checks [11–13]. The DMP for CHD also showed positive trends in mortality reduction, cost development and guideline-based medication [14]. However, currently identified weaknesses in the prevention of cardiovascular and other chronic conditions imply that there is room for improvement in DMPs.

Self-management support (SMS), one of the six core components of the chronic care model, is critical to achieve effective clinical outcomes. It refers to the process of enabling chronically ill people and their families to play a key role in managing their disease and supporting them in making informed decisions and in adopting healthy behaviours [15]. Accordingly, SM involves patients’ active participation in the everyday care of their conditions, as well as maintaining overall health, preventing disease progression and managing the psychosocial consequences of illness [16].

In 2013, SMS, offered by physicians and/or trained nurses in Germany and other European countries, was mostly underutilized [17]. Cramm and Niebor reported that Dutch DMPs should place greater emphasis on patients’ capabilities and empowerment to stimulate their SM, as well as to improve their health and quality of life [18]. A systematic review by Dineen-Griffin et al. demonstrated positive effects of SMS interventions on disease knowledge and control, self-efficacy, clinical indicators and health-related quality of life [19]. Nevertheless, several studies have shown that SMS needs to be more patient-centered [20, 21].

While SMS has been discussed as beneficial in Germany [22], there is little research on how to foster its integration into broadly rolled out DMPs in primary care.

In the German healthcare system, GPs are the central service providers and the first point of contact for most insured patients, treating more than 80% of chronically ill patients. On average, insured persons have about seven contacts per year. With increasing age and disease burden, the number of GP consultations increases to more than 12 per year, including the mandatory DMP appointments (2–4 per year). Hence, GPs usually have a longstanding contact with their patients, often including their relatives. Therefore, it is of central importance to explore what GPs think about or expect from patient SM and interventions to increase SMS in primary care.

The trial “Personalized Self-Management Support Program (P-SUP)”, funded by the Federal Joint Committee (Gemeinsamer Bundesausschuss G-BA; funding number: 01NVF18033; study registration number at the German Register for Clinical Studies: DRKS 00020592, registration date: 16.07.2020), aimed to expand the existing DMPs for T2DM and CHD in Germany through SMS interventions in the areas of physical activity, diet and motivation through peer support [23].

The present study focused on complementing the P-SUP trial with information on providers’ current perspectives on SM in the DMPs for T2DM and CHD. It also aimed to identify strategies to improve SM. Since most patients are enrolled in these DMPs by GPs, the study did not include cardiologists and diabetologists, who also enrol some patients in these DMPs.

While the initial part of the study focused on the general experiences of GPs with the DMPs for T2DM and CHD, in particular their perceived effectiveness, structural components and GPs’ willingness to continue DMP care, as presented in a separate publication [24], this part of the study aimed to answer the following research question:

What are GPs’ experiences with and strategies for supporting self-management of patients enrolled in the DMPs for T2DM and CHD in the North Rhine region of Germany?

## Methodology

### Study design

As the data currently available on the research question are very limited, we chose an exploratory qualitative design to generate information on SMS in the DMPs for T2DM and CHD from the perspective of health care providers. Data were collected through focus group discussions (FGDs) with GPs, following the Standards for Reporting Qualitative Research (SRQR) [25].

FGDs allow for group dynamic processes that promote opinion-forming group discourses [26]. To enable the formation of various interactions and opinions, a minimum of four FGDs were planned, with four to seven participants per group. The use of a semi-structured FGD guide enabled participants to discuss preferred topics in greater depth and to introduce additional topics [27].

### Semi-structured FGD guide

The semi-structured interview guide (see Additional file 1) was developed by the study team based on the topics of the collaborative research project P-SUP. Subsequently, five GPs from the University Hospital Cologne, who had experience in DMP care, reviewed the guides’ structure and content.

Finally, the following topics were defined:

1. Experience with the DMPs for T2DM and CHD
2. Experiences with SM in the DMPs for T2DM and CHD
3. Opinions and attitudes towards planned DMP extension by SMS interventions as in the P-SUP project
4. Dealing with nonadherent DMP patients

### Study inclusion criteria and recruitment of participants

The study inclusion criteria were as follows: GPs working in GP practices in the area of the Association of Statuary Health Insurance Physicians North Rhine (Kassenärztliche Vereinigung Nordrhein [KVNO]) with experience in the care of patients in the DMPs for T2DM and CHD during the study period (definition: ≥ 50 enrolled patients with T2DM and ≥ 20 with CHD). These data were collected via a short questionnaire on GP and practice characteristics developed by the study team to reflect the variance in individual characteristics (see Table 1).

Participants for the FGDs were recruited from the e-mail list of 335 GPs affiliated with the Institute of General Practice at the University Hospital Cologne. This approach was chosen for pragmatic reasons, as experience has shown a higher participation rate among affiliated GPs. Furthermore, a purposive sampling strategy was applied to recruit a balanced sample in terms of age, gender and practice location.

After expressing interest in participating, GPs received the study information, the consent form and the questionnaire on GP and practice characteristics by post or email, and were telephonically contacted by the study team for further information. Prior to participation, participants’ written consent for the overall study, tape recording, data protection and compensation (€80 per person) was obtained. The study received positive ethics approval (ethics approval number: 20-1139) from the Ethics Committee of the University Hospital Cologne.

### Conduction of FGDs

In August and September 2020, 20 of the 25 GPs initially recruited were interviewed in five FGDs, with three to seven participants in each group. A fifth FGD was conducted to check for inductive thematic saturation, as new codes were found in the planned fourth FGD, but did not generate new codes.

The main questions in the interview guide were followed as planned and were not changed during the onset of the study. Due to the familiarity of the group, all the respondents participated actively.

Despite the open orientation of this study, the majority of the statements focused on patients with T2DM, which reflects the larger proportion of patients in the DMP for T2DM.

The FGDs lasted 117 minutes on average (range: 90-126 min).

### Implementation, transcription and analysis

The FGDs were jointly moderated on the premises of the University Hospital Cologne by two interview-trained researchers (LP [health scientist] and LL [health economist]) from the Institute of General Practice of the University Hospital Cologne. The researchers were partly known to the participants but not in a dependent relationship with them. When moderating the FGDs, care was taken to use only a few questions and narrative impulses in order to leave space for open and honest discussions within the group. With the consent of the participants, the FGDs were audio-recorded and transcribed verbatim by a professional transcription office.

**Fig. 1:**
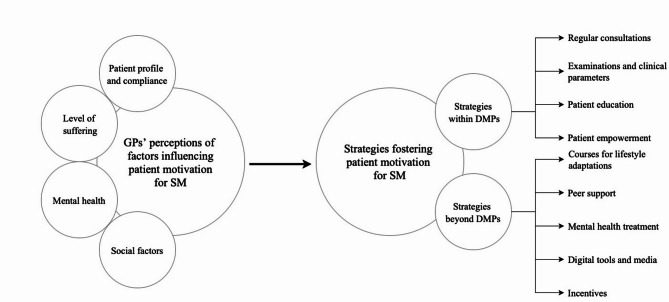
Category system on GPs’ perceptions of factors influencing patient motivation for SM and strategies to foster it

The transcripts were analyzed via Kuckartz’s content-structuring qualitative content analysis by using the software MAXQDA 2020. First, the transcripts of the FGDs were viewed independently by three researchers (LP, LL and KM [social and educational scientist]) and coded according to the deductive categories of the interview guide. Inductive main and sub-categories were then formed based on the codes of the data. Memos were used to record thoughts and ideas for later definitions of inductive coding [28]. After approximately half of the transcripts had been viewed, the categories developed so far were discussed and adjusted by the investigating researchers. These categories were then evaluated and discussed by a multi-professional team consisting of GPs, psychologists, health scientists and an anthropologist to incorporate a broader wealth of experience within the context of the FGDs. Building on this, the categories were further refined and reapplied to the entire category system to broaden and deepen the results. Through the joint coding process and the discursive agreement between scientists from different disciplines, a concise and precise category system was developed (see Fig. 1).

## Results

### Sample description

The average age of the GPs was 56 years. The group consisted of three women and 17 men, with an average of 27 years of professional experience, and characterized by practice ownership. The practice type was evenly distributed among single and group practices, while the practice location was over-represented in urban areas by 13 to seven (see Table 1).

**Table 1:** Characteristics of general practitioners (*N* = 20)

**Characteristics of general practitioners (** ***N*** ** = 20)**	
Age **(Mean [SD; range])**	56 (8.2; 39–69)
Gender (female/male)	3/17
Years of work **(Mean [SD; range])**	27 (9.7; 5–42)
Years of work in resident practice **(Mean [SD; range])**	19 (9.6; 5–38)
Practice owner/employee	19/1
**Characteristics of practices (** ***N*** ** = 20)**	
Practice type (single/group)	11/9
Practice location (urban/small town-rural)	13/7
**Estimated number of DMP patients per quarter (** ***N*** ** = 17)**	
DMP T2DM **(Median [range])**	101–200 (50–600)^1^
DMP CHD **(Median [range])**	20–50 (20–250)^2^

Abbreviations: DMP = disease management program; T2DM = type 2 diabetes mellitus; CHD = coronary heart disease

^1^ Answer categories: <50, 50–100, 101–200, 201–400, 401–600, > 600

^2^ Answer categories: <20, 20–50, 51–100, 101–200,  ≤250, >250

### Main categories of the FGDs

In this publication, we focused on subject areas 2 and 4 of the semi-structured FGD guide (for further findings see [24]). We identified three qualitative main categories:

1. GPs’ perceived SM in the DMPs for T2DM and CHD
2. GPs’ perceptions of factors influencing patient motivation for SM
3. Strategies fostering patient motivation for SM

### GPs’ perceived SM in the DMPs for T2DM and CHD

The first main category included the subcategories “GPs’ understanding of SM” and “GPs’ perceived level of SM in patients enrolled in the DMPs for T2DM and CHD”. 

### GPs’ understanding of SM

When opening the second topic of the FGDs on the perceived SM in the DMPs for T2DM and CHD, some GPs asked for clarification on the definition of SM before entering the discussion. This revealed a variation in participants’ understandings of SM.

Some respondents discussed SM in terms of patients’ health behaviour, particularly in relation to diet and physical activity. Others saw it as an option for patients to measure their blood pressure and/or blood glucose independently by different devices (such as “Freestyle Libre Sensor” and “Accu-Chek”).

SM was also seen as something that applies rather to educated people: “*I have predominantly uneducated patients*,* to put it kindly*,* and this urban-academic striving for self-management*,* preferably digitally supported—is rather zero with uneducated patients*” (I2, FGD5).

In addition, some GPs reported that *“Self-management is a bit of a contradiction to the DMP*,* because in the DMP*,* we bring the patients in to look after them*,* to put them in a passive role*,* so that they listen and learn*,* what they have to do to change their behaviour*,* and on the other hand*,* we now say ‘Okay*,* he should just do it himself’. It seems a bit like someone who accelerates and brakes a car at the same time”* (I5, FGD4).

### GPs’ perceived level of SM in patients enrolled in the DMPs for T2DM and CHD

According to GPs, SM regarding health and lifestyle behaviour was low or non-existent in most of their patients, leading to a discussion about the basis of SM, i.e. individual motivation and/or personal abilities of patients. It was also noted that patients who are able to practice SM are usually not found in DMPs: *“The people we have to motivate are those who do not have intrinsic motivation. Those who do have are not in the DMPs. So self-management is naturally more difficult for them. The people who can self-manage are already doing it. So this is a negative selection of patients in the DMPs”* (I4, FGD 4).

Furthermore, constitutional and institutional conditions that influence patients’ motivation for SM were discussed. According to GPs, most patients were aware of the importance and impact of their behaviour, but lacked motivation to change it. GPs also stated that it takes a lot of effort for GPs and patients to achieve lasting behavioural change: *“You first have to talk about what a lifestyle change means and that it takes at least three months to get used to. You have to create a willingness; It is very tough*” (I4, FGD4).

In addition to the aforementioned topics, the second and third main categories of the FGDs explored “GPs’ perceptions of factors influencing patient motivation for SM” and “Strategies fostering patient motivation for SM”, respectively. While the second category focused on the challenges and barriers to motivation, the third category added strategies to this perspective that could increase patient motivation for SM. With regard to the focus on DMPs, these strategies were categorized into those currently integrated into the DMPs and those that go beyond the actual DMP care, as shown in Fig. 1.

### GPs’ perceptions of factors influencing patient motivation for SM

According to GPs, different personal and social factors influence SM in patients, such as patients’ profiles, compliance with treatment, level of suffering, mental health and social surroundings.

#### Patient profile and compliance

GPs frequently perceived patients with T2DM to be predominantly from working class backgrounds with lower education levels. This resulted in a lower understanding and awareness of the severity of the disease and its consequential conditions, as well as a lower receptivity to GPs’ advice on behavioural change. However, the majority of GPs described their patients’ knowledge of their condition and beneficial health behaviours as sufficient. Nevertheless, GPs found that their patients had only a limited capacity to translate theoretical knowledge into practical applications in their daily lives. Furthermore, these individuals were often described as obese and multimorbid, with a higher prevalence of mental health conditions. According to GPs, they also lacked motivation and exhibited a tendency to engage in dishonest self-perceptions regarding their health behaviours to avoid feelings of shame. Some patients were also believed to have a deeply internalized misconception of healthy eating behaviour from an early age. Although GPs experienced similar attitudes and compliance issues among patients with CHD, there was no prevalent profile attributed to them.

Nevertheless, GPs saw a perceived increase in compliance and a decrease in hospital stays and derailments among patients with T2DM since the implementation of the DMP. Even with generally low and unstable compliance levels, the number of completely non-compliant patients was reported to range from 30 to 50%. One participant noted: *“What is my criterion? The HbA1c or something else? Therefore*,* 90% excuse me*,* 50% participate every now and then. It is always up and down”* (I2, FGD2).

In addition, multimorbid patients with impaired physical abilities and/or obesity experienced more difficulties being compliant: *“Many have joint problems*,* other complaints. So getting over themselves is so difficult”* (I1, FGD2).

Compliance of patients with T2DM was generally perceived to be lower than that of patients with CHD. However, the aforementioned defensiveness towards GP recommendations was also observed in some patients with CHD: *“There are also people who say*,* ‘Give me pills and leave me alone with the rest’”* (I4, FGD4).

A low level of suffering among patients with T2DM was seen as one of the main reasons for lower compliance.

#### Level of suffering

The perceived level of suffering and the associated fear of a condition’s severity were frequently identified as strong motivators for behavioural change among patients. Patients with CHD, in particular, often experienced fear-triggering incidents, such as a heart attacks.

Similarly, patients with CHD who had no previous life-threathening incidents took their condition more seriously when one occurred: “*They are only interested when the first angina pectoris attack occurs*,* that is*,* when they wake up, and before that, I do not truly experience anything in terms of CHD*” (I2, FGD4).

Such fear-triggering incidents were rare among patients with T2DM. GPs commonly stated that most patients with T2DM did not feel their condition, and furthermore had the option of suppressing any forthcoming symptoms by medication, which was not the case for patients with CHD. Thus, there was a high discrepancy between the perceived level of suffering and the actual severity of illness among patients with T2DM, which led them to not take their condition seriously: *“That is the worst thing about diabetes that it does not hurt. Once again: no suffering*,* therefore hopeless”* (I4, FGD2).

Frequently, patients with T2DM only became aware of their condition when complications manifested: *“However*,* the risk is not recognized. It is only perceived when something happens. Until polyneuropathy becomes noticeable*,* until the eyes make a fuss”* (I3, FGD1).

Patients newly diagnosed with T2DM and those who had to start insulin injections often showed a greater motivation for SM at the commencement of treatment. Nevertheless, this motivation diminished over time for patients with T2DM and CHD similarly: “*These are often patients who*,* after a heart attack*,* are motivated to sign up*,* ‘Therefore*,* I’m going to change my life’*,* but after a few quarters you can see that they fall asleep again”* (I1, FGD5).

#### Mental health

GPs reported that psychological disorders are common among patients with T2DM, ranging from depression and anxiety to loneliness: “*So many of these diabetics are depressed*,* very lonely*,* because they are overweight because of social isolation*,* because of illness*,* whatever*” (I2, FGD2).

Moreover, they often identified mental health concerns as the underlying cause of T2DM and, to a lesser extent, of CHD, attributing these conditions to a lack of motivation and physical inactivity throughout life. Conversely, receiving a T2DM or CHD diagnosis, especially in cases of stroke or the need for insulin injections, could cause psychological distress in patients. It was also noted that many patients, specifically from rural areas, were elderly, multimorbid and suffered from age-related conditions, such as late-life depression. Patients with severe mental conditions were particularly prone to treatment derailments: *“Then*,* it is not the classic diabetic. These derailments are often due to compliance issues resulting from mental illnesses*,* as in the case of psychotic patients”* (I1, FGD5).

#### Social factors

GPs experienced misconceptions in the public about T2DM: *“The general opinion is ‘Well*,* my grandma had a bit of diabetes* *too*,* and you can eat a piece of plum cake once a day’. The understanding is not there”* (I2, FGD2).

Moreover, GPs stated that familiar and societal structures had a large impact on the situation of patients: “*Families break up and roots fall apart and that makes people sick* *and frustrated. People are alone; they try to get help somewhere else. That’s what causes all the illnesses*,* and we doctors try to make these people healthy from the outside. That does not work*” (I1, FGD4).

Additionally, GPs reported that patients’ work conditions affected their ability to comply with the treatment, especially in sitting or stressful jobs. Many patients also had financial difficulties, which made them participate less in education and exercise courses. Furthermore, according to GPs, culture and language skills influenced patients’ motivation for SM: *“Everything*,* including attitudes toward the disease. These are completely different cultural groups with completely different ideas about this*,* as we are now imposing on them”* (I1, FGD3).

### Strategies fostering patient motivation for SM

GPs discussed various recommendations and strategies for fostering patient motivation and capabilities in the context of SM. Some of these strategies constituted integral parts of the DMPs, whereas others went beyond the existing DMP care. Hence, we divided the main category into two subcategories, i.e. “Strategies within DMPs” and “Strategies beyond DMPs”.

#### Strategies within DMPs

DMP strategies, which may foster patient motivation for SM, involved four parts: (1) regular consultations, (2) examinations and clinical parameters, (3) patient education and (4) patient empowerment.

#### Regular consultations

According to GPs, regular one-to-one consultations were the core component of DMPs. While GPs stated quarterly consultations as the norm in the DMP for T2DM, they were carried out every three to 12 months in the DMP for CHD.

Many GPs used automatic recall systems to organize appointments and to remind patients, which also increased patients’ proactive engagement in the program: “*We also implemented these recalls via text message or email*,* and many respond to it. They have already made follow-up appointments”* (I1, FGD1).

Furthermore, strict health insurance policies that disenrol patients after two missed appointments, further enhanced regular participation and commitment to the DMP for T2DM.

Consultations were merely used to discuss patients’ current disease state and onset, as well as the associated medical parameters. They were also used to discuss patient behaviours and to increase awareness of behavioural risk factors and possible future outcomes.

While some GPs employed a rather authoritarian communication style with their patients, the majority preferred a more personal, non-hierarchical approach to foster SM: *“These people have thousand other problems*,* economic*,* social and so on. Therefore*,* they have to know that someone is there for them*,* where they can always discuss their problems and let themselves go*,* if they have to manage themselves”* (I1, FGD4).

Furthermore, GPs asserted that consultations should be non-judgemental, especially for patients with T2DM, to avoid withdrawal: *“This standard recommendation ‘stop smoking*,* eat less and walk around more’*,* is on the top edge of the forehead of the standard overweight type two diabetic*,* because he always hears it*,* and is constantly being made to feel guilty or a bit to blame for it*,* and this is why people avoid it”* (I3, FGD1). Constant positive reinforcement was instead experienced as effective for nurturing behavioural change: *“This is certainly a Sisyphean task. You have to nudge it again and again. However*,* I can see success”* (I2, FGD2).

Other GPs posited that an honest presentation of potential adverse health effects was the most effective method of encouraging behavioural change: *“I offer a sugar with it and say ‘If things go on like this*,* then illness*,* lack of independence*,* need of care in old age*,* etc.’ and I say ‘The countermodel is called “Fit in the box”. You may still die between 80 and 90*,* but you are independent or almost until the end.’ Therefore*,* I add a counterdraft to the fear”* (I4, FGD4).

Regarding patients with language barriers, GPs mentioned challenges in maintaining ongoing consultations, documentation, medical treatments and SMS: *“I’m pleased*,* when the few crosses we can make with them can prove a guaranteed success, and that they come at all, and that we can keep it up”* (I1, FGD3).

In cases of serious compliance issues and deteriorating health, GPs referred patients to specialised diabetes care practices and hospitals until their condition stabilised. Having a good network with specialised clinics for fast referrals was seen as pivotal. Furthermore, medical specialists were believed to have more effective, albeit less personal access to patients: *“The ophthalmologist most likely has another chance to get on people’s bad side*,* since they are not reticent anyway in their explanation ‘You will soon go blind’. It is simply much more authoritarian than with us”* (I3, FGD1).

Nevertheless, DMP care did not reach all patients: *“This does not change the fact that people with poorly controlled diabetes and unfavorable overall circumstances are not willing to make lifestyle changes. No DMP is of any use there”* (I2, FG3).

#### Examinations and clinical parameters

GPs believed in a significant benefit from periodic examinations: “*Regular laboratory checks give the patient the opportunity to see something for himself*,* which gains a certain significance over time*,* because he can measure how his own metabolic situation is. This also gives him feedback on what he does with exercise*,* diet and medication. That the whole thing ultimately has positive consequences and it gives us the security that the patients are seen regularly*” (I2, FGD4).

In particular, regular foot and eye examinations were found to be effective preventive measures in the DMP for T2DM. Medical assistants often performed foot examinations due to their closer personal relationship with patients and to save GPs’ time. However, standard examinations in the DMP for CHD were criticised for being ineffective, limited and restricting doctors’ freedom of action.

Apart from regular examinations, GPs frequently mentioned the HbA1c clinical parameter as a specific motivating factor for patients in the DMP for T2DM: *“DMP Diabetes should be viewed differently to the other DMPs*.* One reason for this is quite clearly that it is value-centred*,* with HbA1c as a central value*,* which is also easy for patients to understand. After three appointments at the latest*,* they have become accustomed to it and can always see where they stand”* (I4, FGD2).

The same was observed for insulin-dependent patients using novel devices to measure their blood glucose levels independently: *“The first thing that comes to mind is improved compliance*,* as soon as they no longer have to pick*,* but instead have an Accu-Check”* (I4, FGD3).

Furthermore, GPs used the HbA1c to support the achievement of mutually defined goals: *“They come once a quarter to have their blood taken and for the examination*,* and then we have the opportunity to define goals for the coming quarter and the HbA1c is a motivator”* (I2, FGD1).

There was no such core value in the DMP for CHD: *“The first difference in the management of patients with CHD is that we simply do not have a parameter that we can present to the patient*,* which can be used as a guide and provides a constant benchmark to be followed”* (I3, FGD4).

However, regular blood pressure and weight measurements were also mentioned as motivating values for patients with CHD.

#### Patient education

Educating patients about their illness and favourable health behaviours was a central topic in the FGDs and essential for effective SM. In addition to individual one-to-one consultations, a standardised group patient education program was provided after enrolment in both DMPs. This program was conducted by practice nurses, who were supported by either GPs or diabetologists. However, according to GPs, this program was rated as insufficient to meet patients’ needs. GPs demanded more frequent patient education: *“A short training course once a year would be even more motivating. Refresh your knowledge and simply hear it from someone else again”* (I4, FGD4).

Education courses should also consider patients’ existing knowledge and daily lives, and include suitable, implementable, patient-centered recommendations: “*People want specific tips. They are then grateful*,* ‘Good tip*,* I have implemented it’”* (I4, FGD4).

It was stated that current education programs were designed too far from patients’ reality: *“The problem is that such programs are made by educated people in a metropolitan, upper middle-class environment*,* but the reality*,* at least in my environment*,* is different”* (I2, FGD5).

Besides that, patients with a migration background were unable to access these courses due to language barriers.

According to GPs, there was a lack of specific nutrition courses within DMP care: “*There is no meaningful, non-commercial nutritional advice in our healthcare system. If we manage to obtain institutionalised nutritional advice*,* that would be a gain. It does not exist”* (I2, FGD5).

Nevertheless, GPs rated the current DMP education courses as important for disseminating knowledge and fostering motivation but emphasized the necessity of regular consultations to sustain patients’ motivation: *“It starts with the structured training course, which can then be discussed, ‘What have the patients changed?’ Then, you see the first successes and ask, ‘How is it working?’ This certainly has a big influence*,* but the problem is that, at some point*,* it can fall asleep*,* and then the doctor is needed more and more to educate and motivate them again”* (I1, FGD5).

While most statements focused on patients with T2DM, patients with CHD were also advised and motivated to make behavioural changes, albeit less frequently and over broader consultation intervals.

#### Patient empowerment

Some GPs stated that a shift away from traditional treatment was necessary to increase patients’ responsibility for their own health: *“There has been a paradigm shift in the thinking of many colleagues that patients should be their own best doctor*,* but often, responsibility is handed over at the doctor’s door. My responsibility is to do recommendations*;* the implementation is up to the patient, which is why self-management is a great thing”* (I5, FGD4).

Another reported way of supporting SM was the mutual setting of goals together with the patient. These goals should be implementable, accessible and achievable in patients’ daily lives: *“We just have to set small goals and not overburden them. It is very easy to overwhelm them; if we set our own standards*,* they’re gone”* (I1, FGD2).

GPs frequently emphasised the importance of shared decision-making with their patients. They encouraged this approach by using software, such as the instrument “Arriba”, to demonstrate potential outcomes to their patients. As previously indicated, patients may also be empowered by devices for independent and continuous blood glucose monitoring, enabling them to observe the effects of specific foods and exercises on their glucose levels on an ongoing basis: *“Simply because he sees the necessity himself and immediately feels ‘If I do this*,* this will happen’*,* and his insulin requirement has dropped to less than half”* (I4, FGD3).

To increase patients’ responsibility for their own health, GPs had to accept the decision of patients who did not want to follow their recommendations: *“When I was younger and less experienced*,* I truly annoyed them*,* so that one patient told me ‘I like you and you are a good doctor*,* but when you tell me this again*,* I look for another doctor’”* (I4, FGD4).

### Strategies beyond DMPs

GPs’ recommendations beyond DMP treatment focused on the following subcategories:

1. courses for lifestyle adaptations,
2. peer support,
3. mental health treatment,
4. digital tools and media, and
5. incentives.

#### Courses for lifestyle adaptations

Patients with CHD and other heart diseases have the opportunity to take part in specific cardiac sport groups, which is not the case for patients with T2DM. Consequently, the theoretical knowledge acquired through the DMP could not be transferred into practice. As a result, GPs often emphasized the need for specific and regular exercise courses for patients with T2DM, to which they can be referred to.

Furthermore, GPs recommended that facilities offering exercise courses should be located in central areas that are easily accessible for elderly and/or physically disabled patients. It was also advised that these courses be accompanied by professional guides, such as doctors, medical and sports students, to foster motivation and longevity. As one participant observed: *“It works very well there. People have been in these groups for many years and meet regularly”* (I1, FGD2).

Another recommendation for the longevity of such courses was to affiliate them with well-established local fitness and/or rehabilitation centers, that are connected to public institutions and have experience in insurance and financing matters.

Opinions regarding the group composition of such courses varied. The majority of GPs favoured homogeneity in terms of fitness, education, and migration background: *“The range must not be too wide; otherwise*,* the sporty people will find it boring”* (I3, FGD2).

Some GPs emphasised specific exercise and nutrition programs for obese patients: *“As with the DMP Diabetes*,* there should also be a DMP Obesity. I think that would make a lot more sense. However*,* as I said*,* if we could manage to obtain institutionalized nutritional advice*,* that would be a gain. It does not exist”* (I2, FGD5). GPs working in areas with a high proportion of Turkish and Arabic patients stated the need for women-only training groups.

#### Peer support

Peer support for patients with similar medical conditions was identified as another crucial motivating factor. Participation in regular group exercises with active peers, in particular, was seen as highly motivating: *“I believe that the motivation from fellow patients during sports is much more effective than that from one of us”* (I3, FGD4).

Furthermore, group exercises with fixed timings in easily accessible facilities could enhance regular patient participation. Nevertheless, as previously mentioned by GPs, professional guidance is required for such peer sports groups to succeed in the long term.

GPs also noted that regular exchange among peers with similar medical conditions, as well as commonly set goals such as walking a certain number of steps and/or taking part in a city run, were important sources of motivation for lasting behavioural change and SM. 

However, according to GPs, peers often come together due to a high level of suffering, which is mostly absent in patients with T2DM. Consequently, not all GPs were convinced that peer support groups for patients with T2DM would be as effective as those for patients with CHD, who seek relief from their suffering through self-help and/or peer support.

#### Mental health treatment

In GPs’ experience, successful treatment of T2DM and CHD can only be achieved through a holistic approach that includes mental health treatment in the DMP. However, insufficient treatment options hinder SMS: *“Depression as such is not being treated properly at the moment because you are not receiving appointments. As long as depression is not under control*,* you can do whatever you want*,* and nothing will happen”* (I1, FGD3).

Moreover, patients with lifestyle-related diseases should receive support by therapists who are trained in this field: *“They need to learn to be mindful*,* live normally and recognize their limits*,* and if we can find a psychologist who can explain this properly to the patients*,* we might have a good thing again”* (I3, FGD1).

Specialised treatment options for elderly, multimorbid and/or mentally ill patients were also emphasized to prevent derailments.

#### Digital tools and media

Some GPs shared additional T2DM information with their patients, such as the free online patient information portal “TheraKey”, especially for patients with different language skills: “*I also work a lot with this TheraKey instead of training courses*,* instead of all the stuff that does not work and language categories have not been worked out yet. I now get Turkish patients because it has been translated too”* (I1, FGD3).

Such location- and time-independent information materials were also deemed beneficial for patients with professional and/or family commitments. In general, GPs viewed the upcoming use of digital health applications (DHAs) and online media as advantageous for SM. They recommended integrating apps and media that patients regularly use into routine DMP care to transform current DMP education into a more patient-centred format.

Apart from highlighting digital pedometers as an accessible tool to encourage physical activity by raising awareness of activity levels, fitness apps were identified as a motivational benchmarking tool for peers: *“There are so many fitness apps and watches that work on the principle of comparison. You compare yourself with predefined values via Bluetooth or the internet*,* and this motivation to be better than the rest and to show that is an incredibly powerful tool”* (I3, FGD4).

Furthermore, GPs recommended free online cooking classes and easily usable DHAs for supporting a healthy diet: *“Where you can hold your phone on the food or read the barcode for determining the calories and composition. A change in awareness achieves a lot”* (I2, FGD5).

#### Incentives

Incentives offered by health insurance providers, such as in the form of bonus booklets for the documentation of preventive measures, were reported to be in high demand by patients, with monetary incentives being at the forefront: *“The number one extrinsic motivation is money. When you are looking for a way to motivate people*,* what do you use? You take money. In addition*,* it can be used universally. It is the only thing that works for almost everyone”* (I3, FGD4).

GPs indicated that these bonuses could also be used to increase patients’ motivation for SM, especially in the long term. One potential approach would be to link digital apps and tools to a stratification system. However, some GPs expressed reservations about the efficacy of monetary incentives, citing a lack of awareness or receptivity among patients.

## Discussion

We identified a broad spectrum of challenges with patient SM, as experienced by GPs. First, GPs had different perceptions of SM. Some viewed it as an independent measurement of clinical values. Others saw it as a form of personal responsibility for health-promoting behaviours. However, GPs considered their patients’ health-promoting behaviour in the DMP as rather low, especially for patients with T2DM. This was reflected in the main topic of the FGDs: patient motivation for SM. Most participants in the FGDs doubted that patients could gain the necessary motivation for effective SM on their own due to many barriers, such as personality profiles, mental state, level of suffering and social factors.

These statements are consistent with the findings of reviews on the motivational aspects of SM [29–33]. By accepting responsibility for patients at the front door, they had hindered their empowerment. Furthermore, GPs often perceived patients with T2DM to be from disadvantaged socioeconomic backgrounds with poor social status and mental health. Despite being cared for in a non-judgemental manner, these patients were also regarded as being less motivated and more sensitive to recommendations, which could (un)consciously lead to stigmatization in both, language and behaviour [33]. This tendency was particularly pronounced among more experienced GPs and could lead to demotivating behaviours, contemptuous treatments and inconsistent levels of empathy towards patients, negatively affecting their SM.

Some GPs also exhibited signs of resignation, frustration and hopelessness, particularly when dealing with non-adherent patients. This, in turn, could also have a negative impact [34]. Standardized consultation methods within DMPs, such as motivational interviewing, could mitigate negative impacts on patient motivation for SM [35, 36].

According to GPs, the greatest obstacle to SM, especially for patients with T2DM, was a lack of intrinsic motivation and psychological pressure that only increased when serious complications occurred. In the case of CHD, this was particularly evident after a cardiovascular event. However, mental comorbidities such as depression also negatively affect motivation, as well as the currently insufficient mental treatment options for the provision of holistic care. The future DMP for depression may counteract this problem [4].

GPs mentioned various strategies for increasing patient motivation for SM, including internationally discussed methods such as regular consultations, standardised patient education, behavioural change courses, mental health treatment, peer support, incentives and digital tools. These recommendations on individual aspects can also be derived from international reviews [37–44]. However, out of these recommendations to foster SM, only regular consultation and standardized patient education are part of the DMP thus far.

Furthermore, while GPs positively rated cardiac sport groups for patients with CHD, they criticized the lack of institutionalized sport course for patients with T2DM as a drawback for an effective treatment. As a result, they were only able to offer theoretical information about the importance of physical activities instead of directly referring patients to relevant courses. Such courses could also foster peer support, which was also rated as a helpful way to motivate patients for healthier SM. This has already been discussed in methodologically different reviews [32, 39, 43]. Increasing SMS with the aforementioned instruments within the current DMP structure with a focus on the individual needs of enrolled patients could improve overall SM.

### Limitations

The study was carried out during the first coronavirus wave in Germany. It cannot be ruled out that under the impact of the pandemic, some statements have gained value that would have been different under other circumstances. In addition, only GPs affiliated with the University Hospital Cologne were recruited, which could have resulted in selection bias with respect to possible statements. Furthermore, the ongoing coronavirus pandemic caused a significant number of cancellations and absences, particularly among female participants, leading to an uneven gender distribution in the FGDs. Approximately 60% of the teaching physicians contacted via the mailing list were male, which also led to a greater number of male participants. Furthermore, the number of patients enrolled in the DMPs for T2DM and CHD varied greatly, with the majority enrolled in the DMP for T2DM. Thus, the main impression of the DMPs came from the care of patients with T2DM, which could have influenced statements explicitly aimed at the DMP for CHD. Besides that, the knowledge of the study topic in advance could have influenced GPs’ statements on SM as well.

## Conclusion

GPs displayed a positive attitude towards intensifying the focus on SM in DMP care. However, there was a certain amount of scepticism regarding the effectiveness of SMS interventions since success largely depends on individual factors, such as patient motivation, as well as institutional framework conditions. GPs’ challenges in fostering an effective SM must be countered with a multilevel approach that exceeds current DMP care.

The qualitative results of these FGDs will be quantified in a follow-up survey via an exploratory mixed-methods approach [44–45] to cover the breadth of GPs’ care in the P-SUP study area in the KVNO region.

## Electronic supplementary material

Below is the link to the electronic supplementary material.

Supplementary Material 1

## Data Availability

The datasets generated and analyzed during the current study are not publicly available due to implications of the ethics committee but are available from the corresponding author upon reasonable request.

## Abbreviations

CHD: Coronary heart disease
DHA: Digital health applications
DMP: Disease management program
FGD: Focus group discussion
GP: General practitioner
G-BA: Federal Joint Committee
SM: Self-management
SMS: Self-management support
T2DM: Type 2 diabetes mellitus
