# Publisher Correction: GPs’ experienced challenges and strategies for supporting patient self-management in disease management programs for type 2 diabetes mellitus and coronary heart disease - a qualitative study

**DOI:** 10.1186/s12875-025-03068-6

**Published:** 2025-11-04

**Authors:** Larisa Pilic, Kira Molkentin, Alina Herrmann, Marcus Redaèlli, Lisa Kupsch, Lion Lehmann, August-Wilhelm Bödecker, Beate Sigrid Müller, Stephanie Stock, Stefan Wilm

**Affiliations:** 1 Institute of General Practice, Faculty of Medicine and University Hospital Cologne, University of Cologne, Kerpener Str. 62, Cologne, 50937 Germany; 2 Institute of Global Health, Heidelberg University, Heidelberg, Germany; 3 Institute of Health Economics and Clinical Epidemiology (IGKE), Faculty of Medicine and University Hospital Cologne, University of Cologne, Cologne, Germany; 4 Institute of General Practice, Heinrich Heine University Düsseldorf, Düsseldorf, Germany

**Publisher Correction: BMC Prim. Care 26, 222 (2025)**

10.1186/s12875-025-02896-w

Following publication of the original article [1], the authors reported that the definition of CHD in the abstract section was deleted. The correct statement is highlighted in bold below.

**Incorrect**:

**Background** Effective self-management (SM) is essential for improving health and preventing severe complications in patients with lifestyle-related chronic conditions, such as type 2 diabetes mellitus (T2DM) and **CHD**.

**Correct**:

**Background** Effective self-management (SM) is essential for improving health and preventing severe complications in patients with lifestyle-related chronic conditions, such as type 2 diabetes mellitus (T2DM) and **coronary heart disease (CHD)**.

The original article [1] has been corrected.
