# Supplementary material for: GPs’ experienced challenges and strategies for supporting patient self-management in disease management programs for type 2 diabetes mellitus and coronary heart disease - a qualitative study
Source: BMC Prim Care. 2025 Jul 12;26:222. doi: 10.1186/s12875-025-02896-w (PMC12255095; doi:10.1186/s12875-025-02896-w)
Supplement: Supplementary file 1 — Supplementary Material 1 [file 12875_2025_2896_MOESM1_ESM.docx]

**Additional file 1: Semistructured interview guide**

1. **Welcome and introduction (3 min)**

Welcome all. We thank you for coming and are delighted that you have found the time to participate in this focus group discussion (FGD) on the topic of “Self-management of patients with type 2 diabetes mellitus (T2DM) and/or coronary heart disease (CHD)”.

My name is ________________ and my colleague is ________________.

We both work at the Institute of General Practice at the University Hospital Cologne.

This FGD serves as a preparation and supplement to the NRW-wide research project *Personalized Self-Management Support Program (P-SUP)*, which is funded by the Federal Joint Committee to incorporate innovative forms of care into standard care. The aim of the P-SUP project is to promote the self-management of the abovementioned patient groups and to improve the course of the disease in the long term.

As GPs with DMP contracts, you are familiar with the care of chronically ill patients and are therefore experts in this field. Your experience, opinions and recommendations will provide us with valuable insights that can also support us in the implementation of P-SUP.

1. **Introductions of the participants (3 min)**

I would like to ask you now to introduce yourself briefly and remind you once again that participation in this FDG is voluntary and can be terminated at any time. The FDG will be recorded so that no information is lost. The evaluation of the data will be pseudonymized, i.e., all personal data will be provided with numbers or other keys. The only access to the keys lies with us, the staff of the Institute for General Practice. You will find detailed information on data protection in the study information and consent declaration sent to you.

1. **Communication rules (3 min)**

Before we start the actual FGD, I would like to point out some communication rules:

- There are no right or wrong answers. It is important for us that you express your individual views openly, regardless of what you hear in this group.
- All the information in this FGD will be kept confidential.
- Every participant should take part in the FGD.
- Only one person should speak at a time without interruption.
- Please follow the conversation and do not engage in side conversations.
- Turn off your mobile phone if possible
- When you leave the room, please do so quietly and without comment.
- Note: As moderator, I will remain neutral. We can now start the FGD.

1. **Subject areas (80–105 min)**

**4.1 Experiences with the DMPs for T2DM and CHD (opening question, 20–30 min)**

- *What are your experiences with the DMPs for T2DMP and CHD?*

Additional questions:

- *What is going well?*
- *What could be improved?*
- *Approximately how many patients do you see per quarter?*

**4.2 Experience with SM in the DMPs for T2DM and CHD (20–25 min)**

- *What are your experiences with SM in the DMPs for T2DM and CHD?*

Additional questions:

- *What importance do you attach to the SM of patients with T2DM and/or CHD?*
- *What are the barriers to SM in patients with T2DM and/or CHD?*
- *What are your recommendations for SM in patients with T2DM and/or CHD?*

**4.3 Opinions and attitudes towards planned DMP extensions by SMS intervention as in the P-SUP project (20-25 min)**

- *What are your opinions on the following interventions?*
- Peer support groups (PSGs)
  - *(Explanation of PSG may be necessary)*
- PSG with exercise sessions (core intervention)
  - *What type of exercise intervention would you recommend?*
  - *Should groups be separated (e.g. by mobility etc.?)*
- Nutritional counseling
- Telephone coaching
- Special interactive (P-SUP) internet platform/access to online information/knowledge transfer

Additional Questions:

- *Which intervention do you think would have the greatest impact?*
- *Are there any other interventions that you would recommend?*

**4.4 Dealing with nonadherent DMP patients (10–15 min)**

- *How many patients are rather difficult to reach (approximately in %)?*
- *How can you motivate them?*

**4.5 Open questions and conclusions (10 min)**

- *Are there any important points that have not yet been discussed?*

Thank you very much for your participation. The information gained is highly important for our research project.
